# A Case Report on Atypical Presentation of Metastatic Prostate Cancer

**DOI:** 10.7759/cureus.20943

**Published:** 2022-01-04

**Authors:** Anthony Lyonga Ngonge, Stacy N Amadife, Felix W Wireko, Isaac Ikwu, Vishal Poddar

**Affiliations:** 1 Internal Medicine, Howard University Hospital, Washington, DC, USA; 2 Pulmonary and Critical Care, Howard University Hospital, Washington, DC, USA

**Keywords:** lobectomy, psa, lung nodule, pulmonary metastasis, prostate cancer

## Abstract

The lung is a common site for metastatic cancers such as colorectal and breast cancer but an uncommon site for prostate cancer. The treatment modalities for primary and metastatic lung malignancies differ considerably; therefore, it is essential to distinguish between them. Here, we present a patient with solitary metastatic lung cancer with prostate as the primary source, which was initially considered a primary lung nodule considering his risk factors. The patient later developed other lung nodules and successfully underwent resection of these nodules with no bone involvement at the time. Follow-up computed tomography (CT) of the chest revealed two other new lung nodules and pleural effusion, and magnetic resonance imaging (MRI) of the pelvis showed bone involvement. The patient was started on gonadotropin-releasing hormone therapy with subsequent downtrending prostate-specific antigen (PSA). Although uncommon, prostate cancer can metastasize to the lungs; hence, clinicians must always have a high index of suspicion when a patient presents with a lung nodule, especially with a prior history of prostate cancer.

## Introduction

The lung is a common site of cancer metastasis for many different tumors, commonly breast and colorectal cancer and squamous cell carcinomas of the head and neck [1]. Also, primary lung cancer is prevalent, especially in people with significant risk factors such as smoking. As the management for metastatic and primary lung cancer usually may follow a different algorithm, it is of utmost importance to distinguish early in the disease course primary lung cancer from metastatic cancer, especially in the setting of solitary lung nodule or a limited number of lesions (oligometastatic disease). Prostate cancer usually metastasizes to bone and lymph nodes as the initial sites of metastases. However, atypical presentations have been reported that describe metastasis to the brain, liver, and thorax [2]. We describe one of such atypical prostate cancer metastasis in this case report.

## Case presentation

A 71-year-old male presented to the pulmonary clinic having been referred by his primary care physician to evaluate an incidental lung nodule on a preoperative chest X-ray (CXR) for cataract surgery, given that the patient has chronic obstructive pulmonary disease (COPD). Other significant medical history included active smoking with over 35 pack-years of tobacco, hypertension, chronic kidney disease grade 3A, osteoarthritis, and prostate cancer diagnosed and treated with radiotherapy over two decades. The cancer had been in remission since then. Review of system was negative for anorexia, night sweats, weight loss, back pain, and dyspnea. On examination, the patient appeared well, normotensive, and afebrile. He had normal breath sounds bilaterally with no wheezing or crackles on auscultation. Cardiovascular and other system examinations were unremarkable.

**Figure 1:**
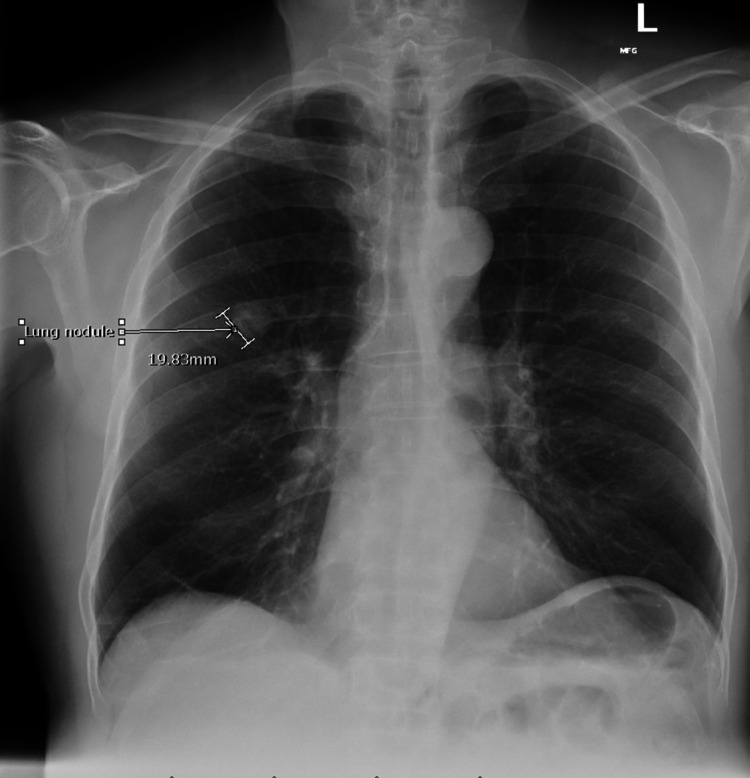
Chest X-ray showing a 2 cm lung nodule

**Figure 2:**
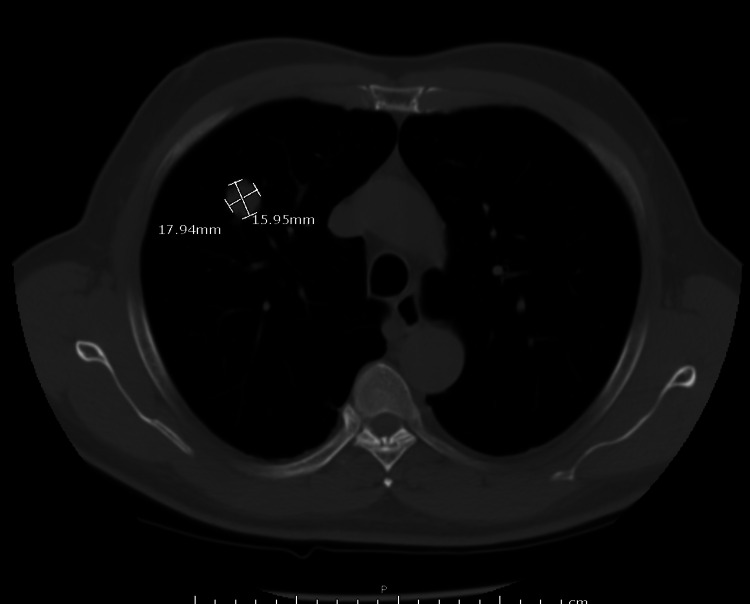
Chest CT scan confirming the presence of an RUL nodule

**Figure 3:**
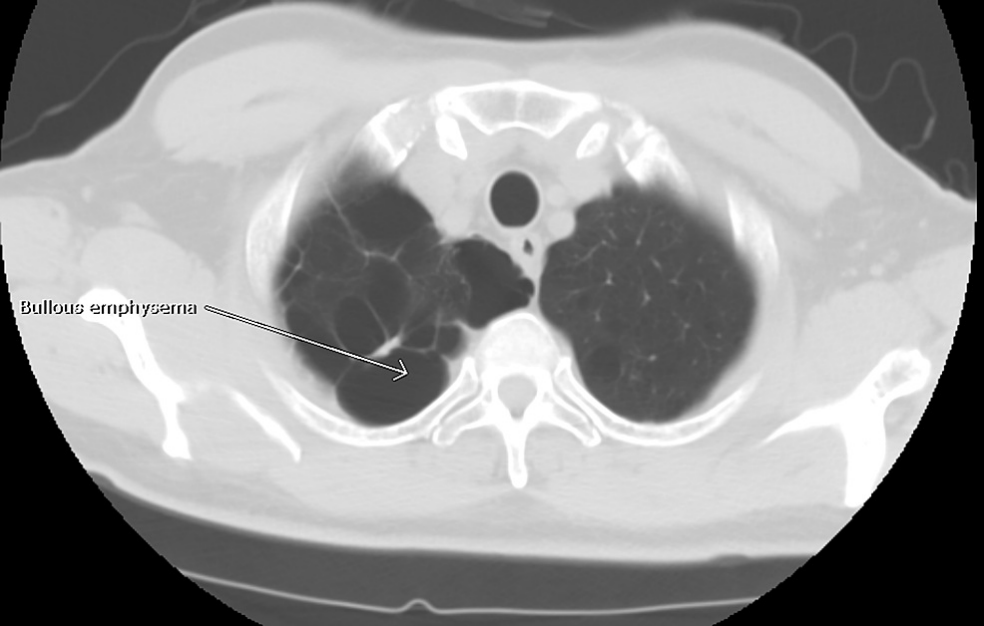
RUL apical bullous emphysema seen on chest CT scan

A review of CXR imaging showed a 2 cm round nodule in the right upper lung (RUL) lobe (Figure 1), with no previous chest imaging for comparison. Primary lung cancer was suspected. A chest/abdomen and pelvic computed tomography (CT) scan was requested to visualize the lesion and help with staging, which confirmed the presence of an RUL round nodule measuring 1.6 × 1.8 cm without any hilar adenopathy (Figure 2). Also noted on the chest CT scan were multiple bilateral lung apical bullous emphysematous lesions (Figure 3). No intrabdominal or bone lesions were identified on this scan. Given the radiological findings and the patient's significant smoking history, a presumptive diagnosis of primary stage 1A lung cancer was made. Transthoracic or bronchoscopic biopsy was considered but deferred as the lesion was located in the periphery, making it difficult to assess without potential injury to the lung.

**Figure 4:**
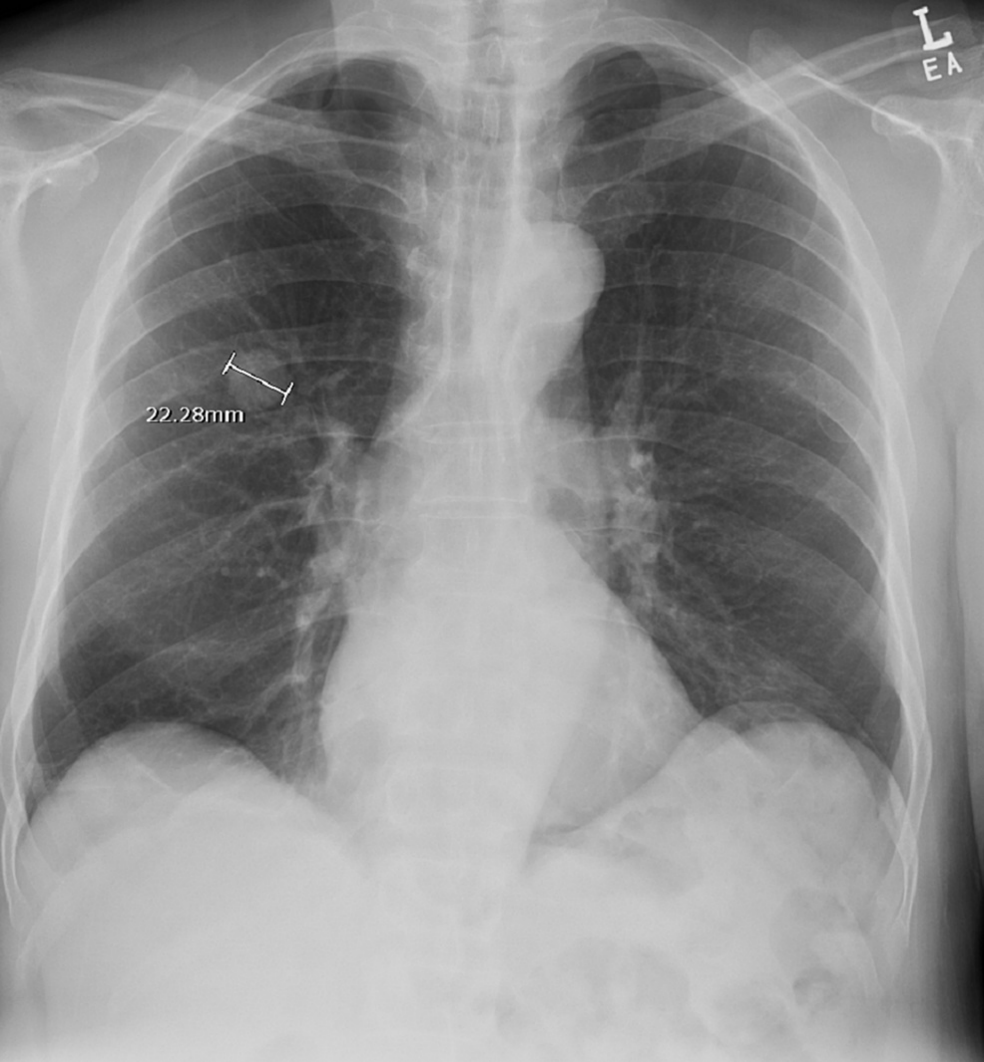
CXR demonstrating increased RUL nodule size to 2.2 cm

**Figure 5:**
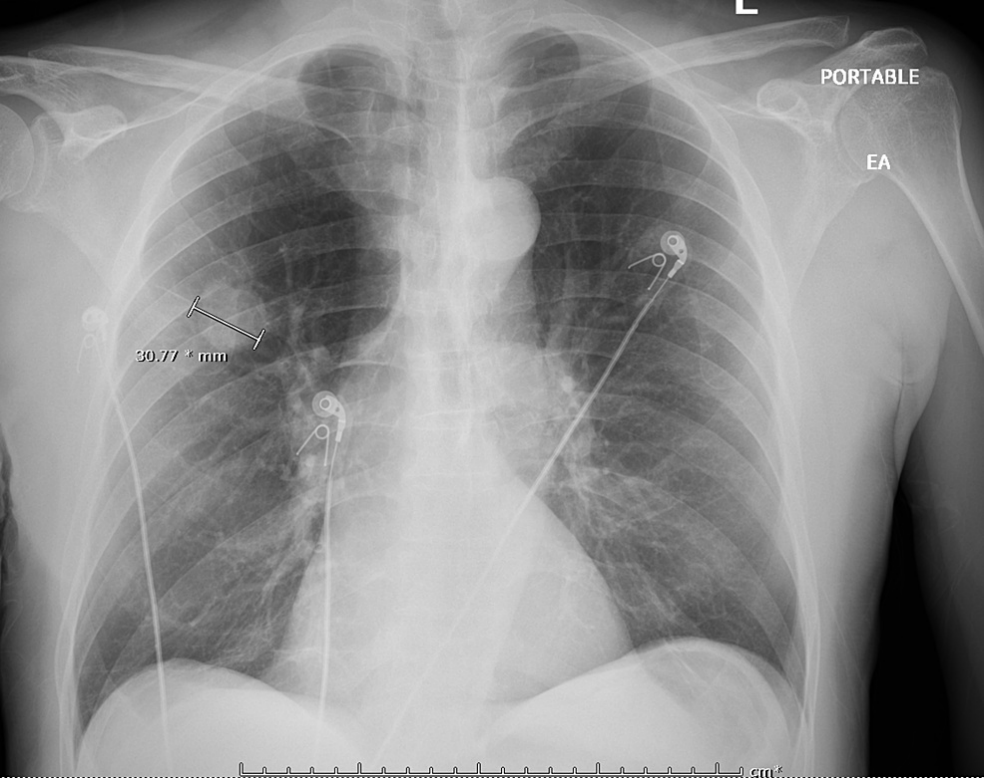
CXR showing increased RUL nodule size to 3.1 cm

**Figure 6:**
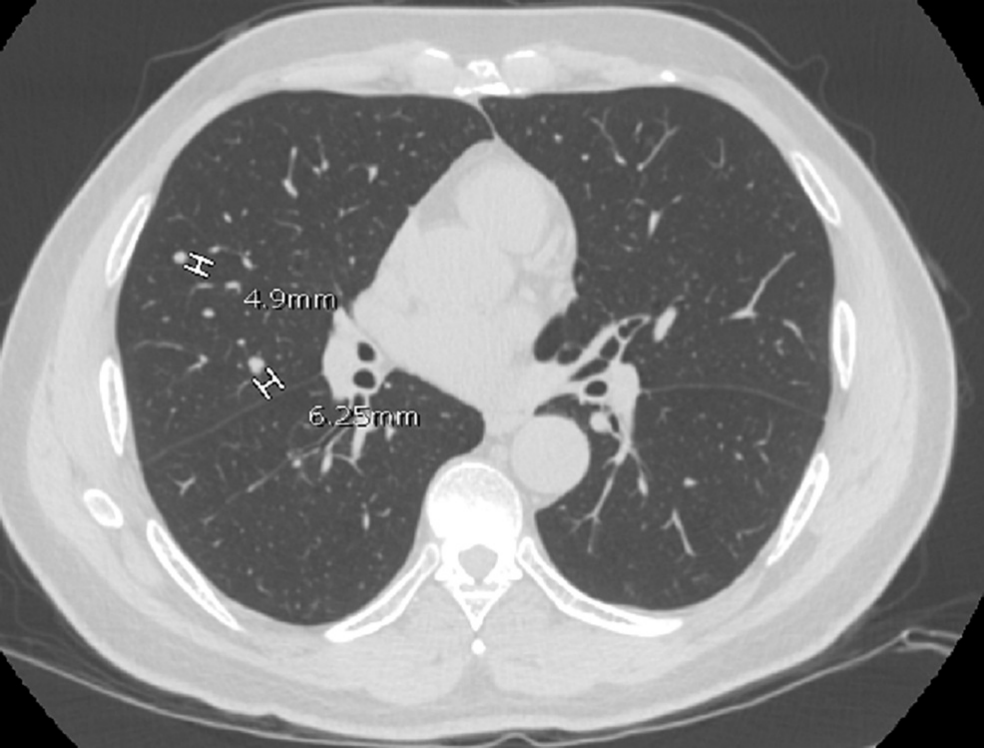
Repeat chest CT scan demonstrating two new RUL nodules

**Figure 7:**
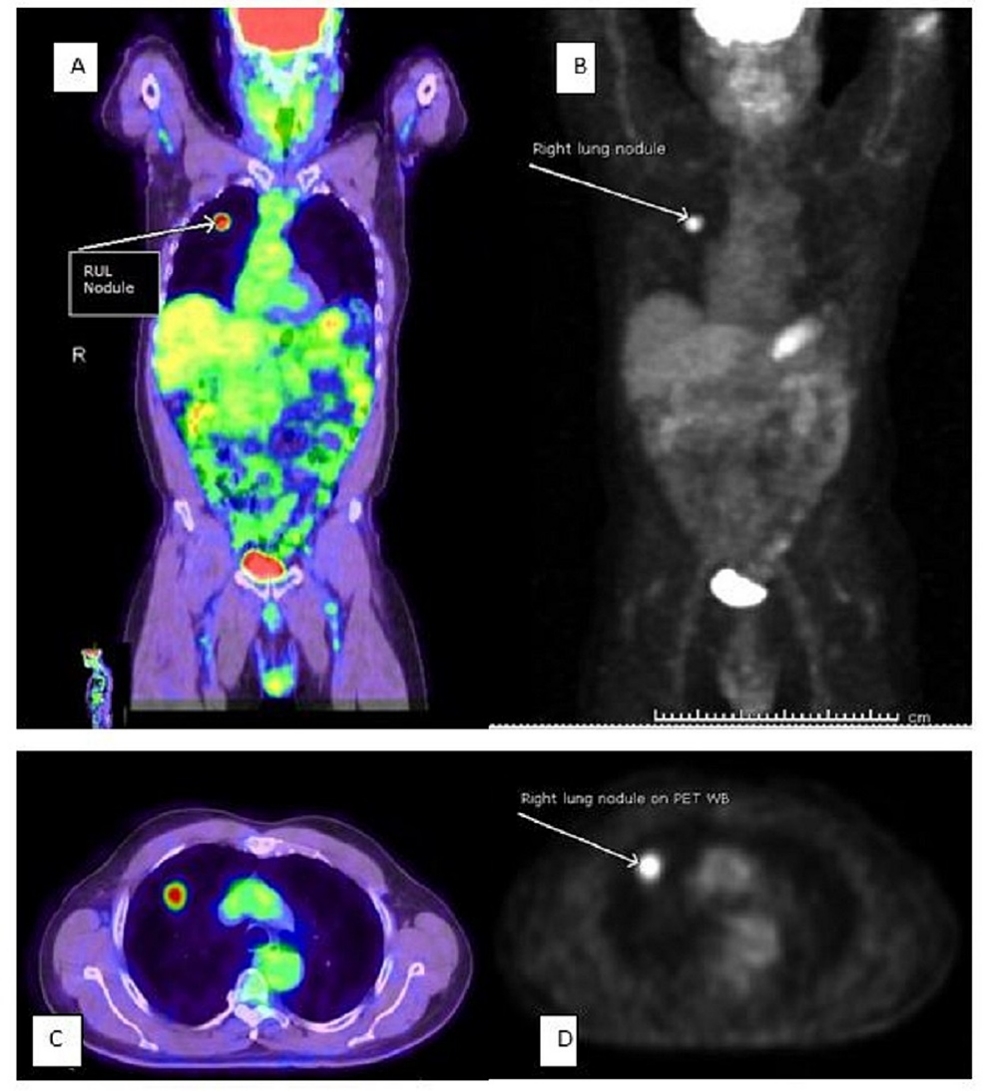
Whole-body PET scan (A–D); A and C depict increased metabolic activity in RUL nodule

A multidisciplinary team comprising a cardiothoracic surgeon, oncologist, patient's primary care physician, and pulmonologist reviewed the case and decided that the patient would benefit from a right upper lung lobe segmentectomy. However, the patient was reluctant to pursue surgery and was steadfast on trying herbal medications despite counseling. The patient failed to show up in the clinic for his follow-up appointments until three months later, where a repeat CXR showed increased nodule size to 2.2 cm (Figure 4), and the patient was still asymptomatic with unremarkable physical examinations. The patient still was reluctant to consider the surgical option. After this clinic visit, the peak of the COVID-19 pandemic came, where clinics were transitioned to telehealth, and the patient did not keep up with his telehealth appointments for another six months. Finally, the patient was successfully reached via telephone and presented for a follow-up clinic visit where another CXR was done and showed increased nodule size to 3.1 cm (Figure 5) and a CT scan demonstrated two smaller new nodules in the RUL measuring 4.9 and 6.3 mm (Figure 6). A whole-body positron emission tomography (PET) scan was done that showed a 2.8 cm right upper lung mass with an 8.3 standard uptake value (SUV) (Figure 7). The two new smaller nodules were not captured by the PET scan. Again, no abdominal or bone lesions were captured by the whole-body PET scan.

Complete blood count, metabolic panel, and prostate-specific antigen (PSA) were within normal limits, except for elevated serum creatinine of 1.42 mg/dL. The patient now agreed and consented to surgery after further counseling.

**Figure 8:**
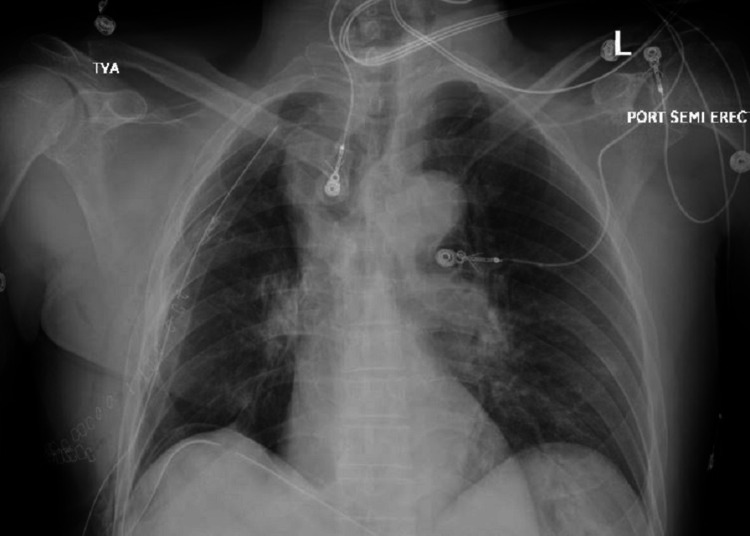
CXR post RUL lobectomy void of any lung lesion

The patient underwent a successful right upper lung lobectomy without postoperative complications. Postoperative CXR was void of any lung lesion (Figure 8). The patient was referred to pulmonary rehabilitation at the time of discharge. Pathology analysis of the resected lung tissue showed that all three lung nodules were metastatic prostate cancer.

**Figure 9:**
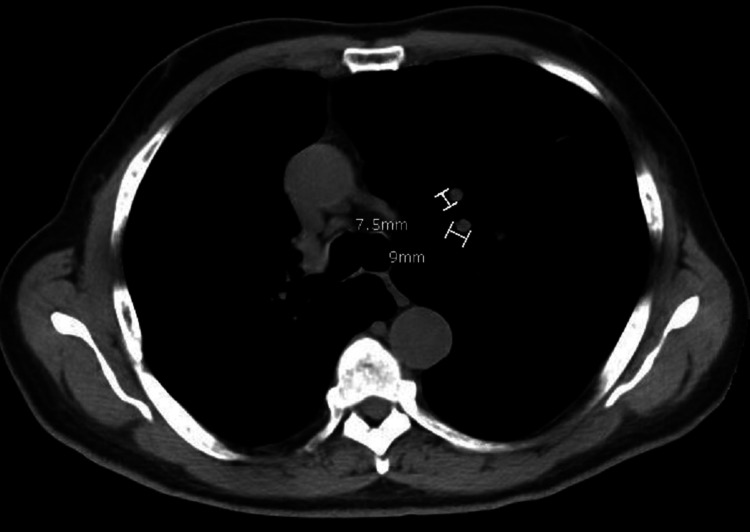
Chest CT scan demonstrating two new left lung nodules

**Figure 10:**
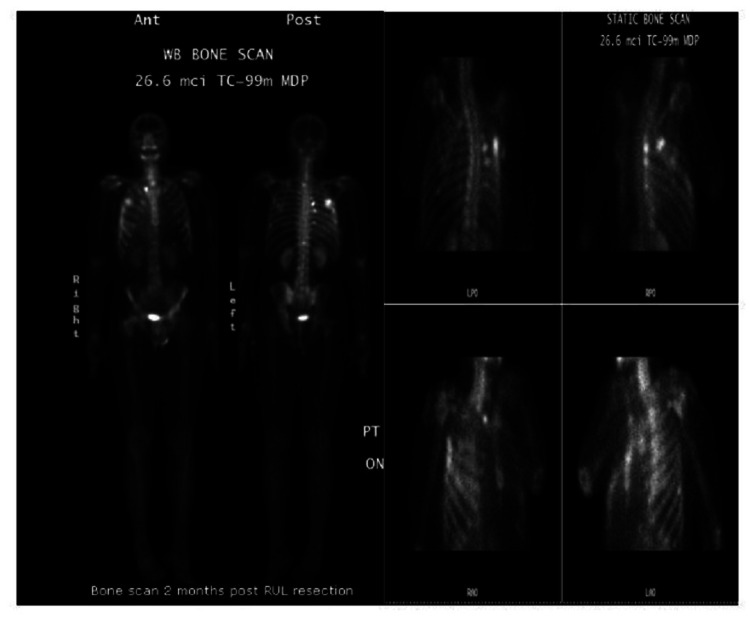
Bone scan demonstrating abnormal increased tracer uptake in right-sided ribs post-surgery likely due to bone healing

CT scan of the chest, abdomen, and pelvis conducted two months post-surgery revealed two new nodules in the left upper lobe and lingula lobes, respectively, and a small right-sided loculated pleural effusion (Figure 9) with no evidence of bony or intra-abdominal metastasis. The patient remained asymptomatic, except for mild pain at the surgical site. Following the CT scan findings, a bone scan was done, which showed multiple foci of abnormal increased tracer accumulation in the right upper ribs, likely due to healing post-surgery; however, we could not exclude metastatic bone lesions (Figure 10). Serial PSA testing conducted at two, four, and seven months post-surgery were 1.38, 6.73, and 31.08 ng/mL, respectively. Given his rising PSA and the new lung lesions, a magnetic resonance imaging (MRI) of the pelvis was done, which showed atrophic prostate with loss of provisional zone of differentiation. The patient was started on gonadotropin-releasing hormone inhibitor, leuprolide and antiandrogen, and bicalutamide for medical castration, which was followed by a significant drop in testosterone levels from 262 to 30 ng/mL and PSA level from 31.08 to 15.1 ng/mL within six months of therapy. The patient has been stable but complains of mild fatigue and dyspnea on moderate exertion.

## Discussion

In light of our patient's significant smoking history and PET scan characteristics of his lung lesions, lung cancer was our initial working diagnosis and not prostate cancer. Prostate cancer has been extensively studied, and its usual initial site of metastasis is to the bone, especially the spine and adjacent tissues [3]. Our patient had an atypical presentation with metastatic prostate cancer to the lung, which was thought to be in remission following radiotherapy over two decades prior.

There are few case reports of atypical metastasis of prostate cancer in literature, including intra-abdominal neuroendocrine metastases [4] and metastasis to the cranial cavity mimicking meningiomas but with small cell neuroendocrine tumor histology [5] and to the pleura mimicking mesothelioma [6]. In 1984, Saitoh et al. autopsied 1885 patients with prostate cancer and found that 49% of 1367 patients with metastatic disease had pulmonary metastases. However, only four of these patients (0.3%) were found to have isolated pulmonary metastasis [7]. Wallis et al. in 2011 found that only 33 clinical cases of isolated pulmonary metastasis have been reported in the literature [8]. These findings, along with a few others, corroborate the rarity of this atypical prostate cancer presentation, which, without pathology analysis, can be misdiagnosed as primary lung cancer, especially when presenting as a solitary lesion in patients with risk factors for lung cancer just as in our patient.

There are two potential mechanisms for the metastatic dissemination of prostate cancer: metastases originating from the primary tumor and cascade of processes in which metastatic lesions metastasize, leading to new lesions [7,9]. In our patient, it is unclear if the two smaller new right upper lung nodules prior to lobectomy were from the initial solitary pulmonary nodule or the primary tumor in the prostate gland. However, the development of new lesions after right upper lobectomy favors metastases from the primary tumor.

Surgical excision is strongly advocated for managing solitary nodule or oligometastatic lesions in patients who are surgical candidates as this may result in a cure or slow the disease progression, especially in the latter mechanism of spread [10]. Falling PSA levels post-resection is an excellent prognostic marker indicating control of the disease. This can be achieved via resection of the metastatic lesion only or with androgen suppression therapy [11]. In our patient, we noticed a rising PSA level months after he underwent lobectomy, which heightened our suspicion of active recurrence of his primary prostate cancer. Although his MRI pelvis showed a shrunken prostate gland, he now had two new left lung nodules that were not evident on prior images indicative of new metastasis and right-sided pleural effusion concerning possible pleural metastasis. Hormonal therapy with androgen suppression is a significant cornerstone of prostate cancer management. This is usually achieved through bilateral orchiectomy or with gonadotropin-releasing hormone analogs. Our patient was managed with the latter using leuprolide and bicalutamide, which resulted in a significant drop in the patient's serum PSA and testosterone levels.

It is actually unclear if the incidence of isolated prostate cancer metastasis to the lung is currently on the rise as we also noticed that in the past year, two similar cases were reported by Yoshitake et al. and Kosaka et al. [12,13]. However, the cases reported by Yoshitake et al. and Kosaka et al. showed that pulmonary metastasis was identified in their patients seven years and 1.5 years, respectively, after completing treatment for primary prostate cancer. Our patient was referred to us due to an incidental nodule seen over two decades after radiotherapy. This may imply that although the recurrence of prostate cancer or metastasis to distant sites may occur within the first five years posttreatment, just as in many other cancers, it can very well occur even many years after. As such, these patients should always be considered as high risk and should have a low threshold for full investigations early enough whenever a suspicious nodule is identified on their imaging.

## Conclusions

Our patient's presentation and evolution reiterate that metastatic prostate cancer can present with a variety of signs and symptoms or be found incidentally on imaging depending on the site of metastases. Hence, clinicians must always have a high index of suspicion, especially in patients with prior history of prostate cancer. Also, it is not uncommon for patients with solitary or oligometastatic pulmonary disease to present with recurrent pulmonary/metastatic lesions after surgical resection, just as in our patient. Thus, patients who are surgical candidates must be informed of the possibility of tumor recurrence prior to surgical resection.
